# Enteroptosis and Neurasthenia

**Published:** 1905-02-25

**Authors:** 


### ENTEROPTOSIS AND NEURASTHENIA.

Dr. Hugh McCallum[1] deals (under the hybrid title Viscero-ptosis, which we take leave to amend) with a common symptom of what is called the "American disease," neurasthenia. Unfortunately for us there is no racial monopoly in a disease which appears to an almost alarming extent in all the civilised communities of the world. It is accepted that abnormal mobility of the viscera is compatible with perfect health, and that such a condition has no inherent symptomatology apart from that with which it is endowed by the exaggerated sensibilities of the neurasthenic. On the other hand the author states that over 90 per cent. of neurasthenic women are the subjects of abnormal mobility of the viscera. The important ætiological factors in the production of the condition are loss of tone in the abdominal musculature, and absence of fat, and both these are prominent features in the bodily condition of the neurasthenic.

Briefly stated, the author's advice is as follows:—It is manifestly idle to attempt to cure by fixation of any one particular organ on whose mobility the attention of the patient has been concentrated, a general condition involving to a greater or less degree all the viscera of the belly. The only proper treatment is some form or other of the Weir-Mitchell method, and the following is recommended: (*a*) Recumbency in bed, as far as possible without pillows, for from four to eight weeks; (*b*) some form of cool bath one hour before massage to secure muscular relaxation; (*c*) general massage; (*d*) raising of the thorax, "to enable the viscera to find room upward"; (*e*) feeding, to secure a packing of fat for the abdominal viscera; (*f*) training of the mind by means of an intelligent nurse, and of the body by massage and a succession of graduated exercises.

Rest-cures and Weir-Mitchell treatments have such an immense vogue at the moment that it may be not out of place to venture a few comments on their application. It is unquestionable that in neurasthenics whose "break-down" is directly traceable to a period of stress, either mental or bodily, the passive exercise, rest and high feeding, which make up the "rest-cure" are of the utmost service, and the basis of the treatment is rational, for the latter in this instance consists of the direct antidotes of the determining causes of the illness. But there is another class of neurasthenics in whom the temperamental factor preponderates, and the origin of the condition is far more obscure. Such patients are sometimes far from being bettered by the accepted rest-cures. Endowed, perhaps, with an unusual faculty for introspection, they find in the solitude and seclusion of their retirement a free field for the exercise of this talent, and may verge perilously upon melancholia. Others again acquire what may be almost called obsessions, not infrequently evidenced by a dread of returning to the locality which witnessed the beginnings of their ill-health. Such a patient may have reached a state of practical cure, yet on the suggestion of return to her old locality or occupation may relapse to an astounding degree. It is useless to persevere with rest-cures in such a case. If the physical health be good, the cure for the obsession is more likely to be found in persuading the patient to a defiance of her morbid dreads, than in a continuation of methods likely to confirm her in her too-readily-accepted conviction that she is doomed to a perpetual invalidism.

[1] Brit. Med. Jour., Feb. 18, 1905.

